# Recent Advances in the Use of Focused Ultrasound for Magnetic Resonance Image-Guided Therapeutic Nanoparticle Delivery to the Central Nervous System

**DOI:** 10.3389/fphar.2019.01348

**Published:** 2019-11-13

**Authors:** Delaney G. Fisher, Richard J. Price

**Affiliations:** Department of Biomedical Engineering, University of Virginia, Charlottesville, VA, United States

**Keywords:** focused ultrasound, nanoparticle, central nervous system, brain, drug and gene delivery

## Abstract

Targeting systemically-administered drugs and genes to specific regions of the central nervous system (CNS) remains a challenge. With applications extending into numerous disorders and cancers, there is an obvious need for approaches that facilitate the delivery of therapeutics across the impervious blood-brain barrier (BBB). Focused ultrasound (FUS) is an emerging treatment method that leverages acoustic energy to oscillate simultaneously administered contrast agent microbubbles. This FUS-mediated technique temporarily disrupts the BBB, allowing ordinarily impenetrable agents to diffuse and/or convect into the CNS. Under magnetic resonance image guidance, FUS and microbubbles enable regional targeting—limiting the large, and potentially toxic, dosage that is often characteristic of systemically-administered therapies. Subsequent to delivery across the BBB, therapeutics face yet another challenge: penetrating the electrostatically-charged, mesh-like brain parenchyma. Non-bioadhesive, encapsulated nanoparticles can help overcome this additional barrier to promote widespread treatment in selected target areas. Furthermore, nanoparticles offer significant advantages over conventional systemically-administered therapeutics. Surface modifications of nanoparticles can be engineered to enhance targeted cellular uptake, and nanoparticle formulations can be tailored to control many pharmacokinetic properties such as rate of drug liberation, distribution, and excretion. For instance, nanoparticles loaded with gene plasmids foster relatively stable transfection, thus obviating the need for multiple, successive treatments. As the formulations and applications of these nanoparticles can vary greatly, this review article provides an overview of FUS coupled with polymeric or lipid-based nanoparticles currently utilized for drug delivery, diagnosis, and assessment of function in the CNS.

## Introduction

As advances in medicine continue to extend lifespans, the prevalence of diseases of the central nervous system (CNS) in the aging population also continues to grow. In turn, this rise in CNS diseases introduces more urgency to improve the delivery of therapeutics to the brain. The brain is protected by the blood–brain barrier (BBB) which precludes most systemically-administered agents from entering its parenchymal space. This exclusion of therapeutic agents restricts treatment options and has greatly hindered successful therapeutic developments for CNS diseases. In addition to challenges associated with surpassing the BBB, the treatment of many CNS diseases also requires spatially targeted delivery of therapeutics to avoid altering normal tissue function. For example, many neuromodulatory drugs will stimulate differential effects depending on the brain region upon which they act. In such cases, non-specific delivery can induce overall dysregulation. In the case of brain tumors, toxic chemotherapeutics may be deleterious if administered to healthy brain tissue in high concentrations.

**Figure 1:**
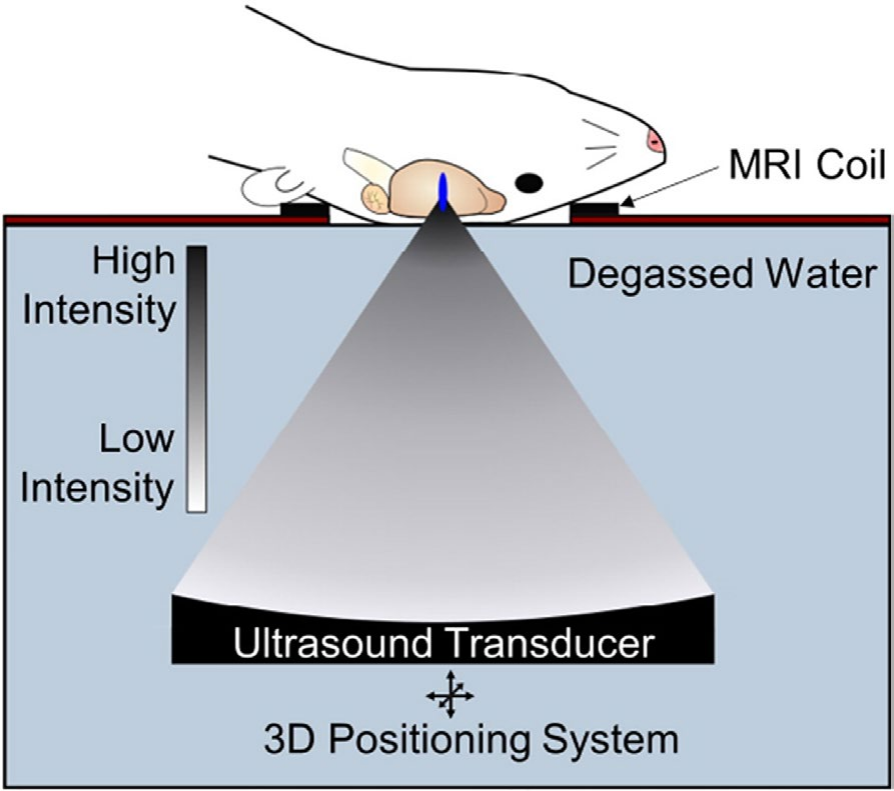
Transcranial focused ultrasound (FUS) with microbubbles yields non-invasive, safe, repeated and targeted BBB disruption, leading to improved drug or gene delivery to the brain. Pre-clinical FUS studies in animals including mice and rats permit use of a single-element FUS transducer, due to favorable skull geometry. FUS can be guided with MR imaging and is capable of sub-millimeter resolution allowing precise targeting of structures in the CNS with minimal off-target effects. Adapted from Timbie et al. (2015). Reproduced with permission.

**Figure 2:**
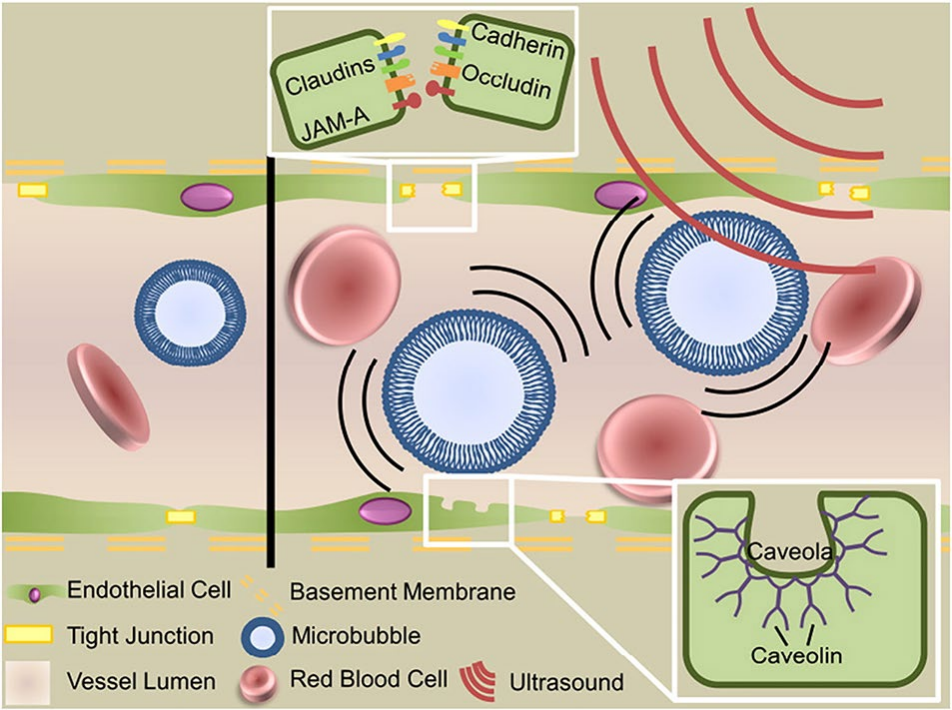
Mechanisms of focused ultrasound mediated blood–brain barrier disruption. Circulating microbubbles oscillate in the ultrasonic field, producing forces that act on the vessel wall to generate three bioeffects that permit transport across the blood–brain barrier: disruption of tight junctions, sonoporation of the vascular endothelial cells and upregulation of transcytosis. Adapted from Timbie et al. (2015). Reproduced with permission.

**Figure 3:**
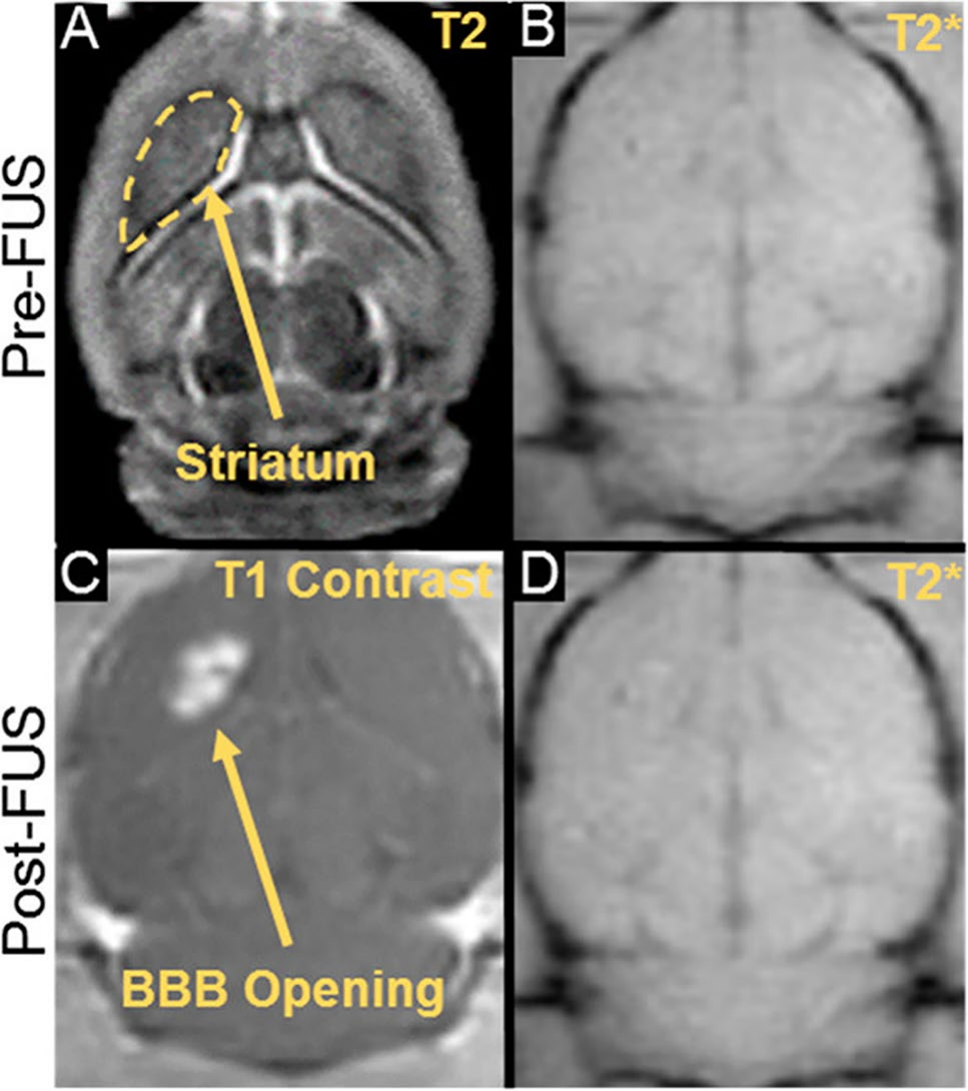
MR imaging for guidance, confirmation, and safety evaluation of FUS treatments. **(A)** Pre- treatment planning using T2 pre-FUS images. **(C)** BBB opening in the striatum as confirmed by post-FUS contrast-enhanced T1 imaging. **(B, D)** Treatment safety may be assessed by comparing pre- and post-FUS T2* images. Adapted from Mead et al. (2017). Reproduced with permission.

One emerging approach to opening the BBB in a spatially targeted brain region utilizes magnetic resonance image-guided focused ultrasound (FUS; **Figure 1**). Several clinical trials demonstrating the efficacy of this technique to safely open the BBB are underway with promising preliminary results (Carpentier et al., 2016; Lipsman et al., 2018; Idbaih et al., 2019; Mainprize et al., 2019). The underlying premise for BBB opening with focused ultrasound is provided in detail in other review articles (Timbie et al., 2015; Curley et al., 2017; Gorick et al., 2018), and we refer the reader to these reviews for a more in-depth report. Briefly, FUS transmits pressure waves that converge on a selected focal spot with millimeter precision. These pressure waves oscillate gas-encasing microbubbles that are administered systemically during FUS treatment (**Figure 2**; Timbie et al., 2015). This mechanical effect leads to the disruption of tight junctions of endothelial cells in the focal region. This disruption then allows systemically-administered therapeutics that are normally obstructed by the BBB to enter the FUS-targeted brain region (Konofagou, 2012). Magnetic resonance image-guidance allows for confirmation of enhanced permeability of the BBB at the desired target as well as monitoring of heating *via* magnetic resonance thermometry (**Figure 3**; Mead et al., 2017). FUS also has the benefit of being minimally invasive in comparison to alternative technologies used to treat CNS disorders. Indeed, both convection-enhanced delivery and deep brain stimulation require invasive interventions. While intranasal administration can noninvasively bypass the BBB, it has limited capacity to selectively target brain regions, is limited by the dosage volume that can be administered, and is difficult to obtain proper alignment in the nasal cavity for effective delivery (Agrawal et al., 2018; Gänger and Schindowski, 2018). Chemical agents (e.g. Cereport and Regadenoson) that modulate tight junctions between endothelial cells have also been proposed. However, these drugs do not provide selective BBB opening and have not yet proven to be highly effective in clinical trials (Prados et al., 2003; Jackson et al., 2017; Jackson et al., 2018).

Transpassing the blood–brain barrier alone, however, may not always be sufficient for efficacious therapeutic treatment. The brain parenchyma contains extracellular matrix components that form a dense, mesh-like structure which further hinders dissemination of therapeutic agents within a target brain tissue (Mastorakos et al., 2015). To overcome this additional hurdle, nanoparticles with strategic surface modifications can allow a therapeutic agent to diffuse throughout the desired brain region (Kenny et al., 2013; Saraiva et al., 2016). There are countless nanoparticle formulations, but in general they consist of a core region wherein a polymer or lipid material encapsulates or presents on its surface the therapeutic agent. The core region is then typically coated with a non-adhesive molecule (commonly polyethylene glycol) and/or molecules intended to bind to specific molecular targets. Such nanoparticle coatings may allow them to more effectively diffuse through a larger volume of brain parenchyma and/or enable them to more precisely bind to specific molecular targets (Suk et al., 2016). Moreover, nanoparticles may be designed to tailor the pharmacokinetics of the loaded drug by improving the therapeutic window, increasing selectivity of dispensation, and/or improving temporal control (Kolhar et al., 2013; Timbie et al., 2017; Zhong et al., 2019).

In this review, recent advances in the use of FUS and nanoparticle design for delivery to the brain are discussed. We begin by reviewing polymers and lipid-based compositions that are commonly used in fabricating non-viral nanoparticles and then follow with discussions of how such nanoparticles are being used in combination with focused ultrasound for therapy, diagnosis, and assessments of function. Emerging developments and prospective areas for research are also explored. We affirm that the combination of FUS and nanoparticles offers promising treatment and detection options for a host of CNS disorders as well as functional study of the brain.

## Polymer and Lipid-Based Nanoparticles for Delivery to the CNS With FUS

### Polymer Components of Nanoparticles for Delivery to the CNS With FUS

#### Polyethylene Glycol (PEG)

**Figure 4:**
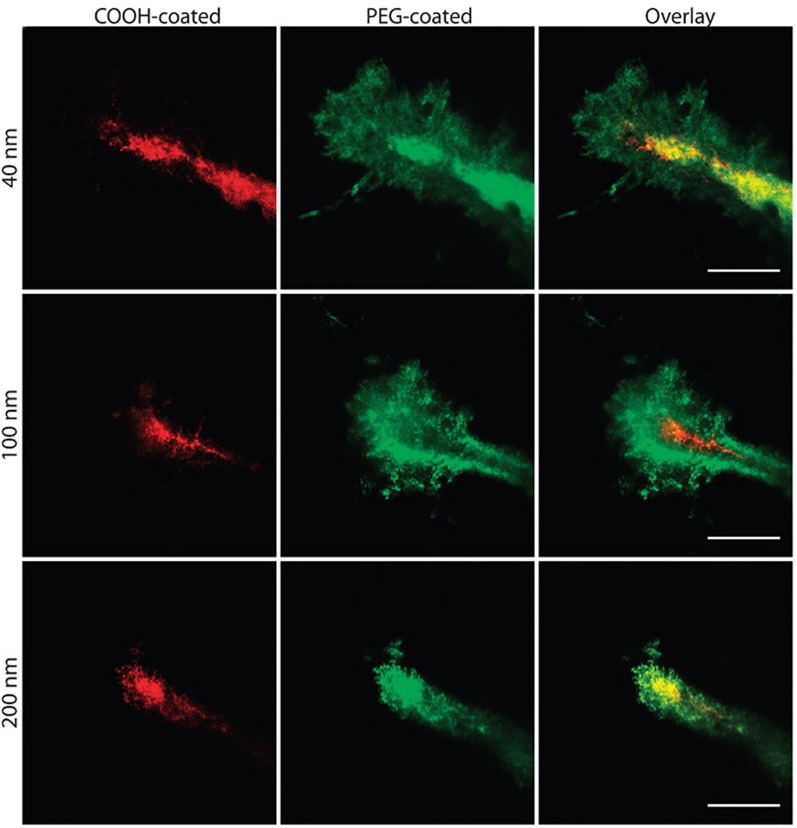
Nanoparticle penetration into mouse brain tissue *in vivo*. Direct comparison of the distribution of fluorescent nanoparticles of similar sizes with different surface coatings after intracranial co-injection into mice. Images were acquired 60 min after injection. Scale bars = 50 μm. Adapted from Nance et al. (2012). Reproduced with permission.

The ability of a nanoparticle to escape detection by immune cells increases its ability to reach the intended target tissue and accumulate there. Nanoparticles may be extracted from the circulation after intravenous administration *via* the reticuloendothelial system. Several proteins are known to bind to nanoparticles (e.g. albumin, immunoglobulin G, apolipoproteins, fibrinogen), which can further hinder the ability of a nanoparticle to reach its target tissue (Soppimath et al., 2001; Aggarwal et al., 2009). To decrease protein adsorption, and thus avoid recognition by the immune system, polyethylene glycol (PEG) is often conjugated to the surface of nanoparticles. Dense coatings of this hydrophilic and flexible molecule can sterically hinder proteins from adsorbing to the surface (Vasir and Labhasetwar, 2007; De Jong, 2008; Aggarwal et al., 2009; Spicer et al., 2018). PEGylation can mask the underlying properties of the nanoparticle core surface, effectively increasing its biocompatibility and half-life (Owens and Peppas, 2006). This being said, there have also been indications that repeated injection of PEGylated nanoparticles can elicit an immune response that leads to accelerated blood clearance of these nanoparticles (Dams et al., 2000). Nevertheless, applying a dense PEG coat to nanoparticles has shown to be effective in producing biocompatible brain-penetrating nanoparticles upon FUS application without producing any significant signs of toxicity (Mastorakos et al., 2015; Mead et al., 2016; Suk et al., 2016; Mead et al., 2017) (**Figure 4**). Further, increased PEGylation yields increased distribution within the target tissue (Mastorakos et al., 2015; Suk et al., 2016; Negron et al., 2019). For example, increasing the PEG density beyond conventional PEGylation ratios when designing PEGylated polyethylenimine (PEI) nanoparticles (i.e. PEG : PEI molar ratio of 26 rather than 8) showed higher brain distribution and gene transfection after injection of reporter gene-loaded PEG-PEI nanoparticles into the striatum of healthy rat brains (Mastorakos et al., 2015). Incorporating PEGylated nanoparticles with FUS allows widespread drug delivery within target brain regions (Nance et al., 2014; Mead et al., 2016; Suk et al., 2016).

#### Poly(Lactic-co-Glycolic Acid) (PLGA)

Poly(lactic-co-glycolic acid) (PLGA) is one of the more widely used core materials for generating biodegradable nanoparticles (Danhier et al., 2012; Panyam et al., 2002). Its constituents—lactic acid and glycolic acid—are readily metabolized, and PLGA has approval for several medical applications by both the Food and Drug Administration and the European Medicine Agency (Danhier et al., 2012). PLGA nanoparticles infiltrate cells within minutes of exposure *in vitro* and are capable of being both phagocytosed and inducing endolysosomal release (Panyam et al., 2002). If introduced to the circulation alone, the hydrophobicity of PLGA triggers the reticuloendothelial system to clear these particles from the blood stream (Owens and Peppas, 2006). Commonly, PEG is conjugated to PLGA nanoparticles to mask their hydrophobicity and prevent plasma protein binding, as described above. PEGylation also helps conceal the natural negative charge of PLGA particles for enhanced uptake of the nanoparticles in *in vivo* conditions wherein a negative charge can cause undesirable protein interactions (Danhier et al., 2012). PLGA nanoparticles are conducive to various surface modifications and drug-loadings allowing successful implementation in a host of applications. These include cancers, inflammation, and CNS diseases (Danhier et al., 2012). Despite successful implementation, PLGA nanoparticles suffer from low drug loading and high drug burst release, which can greatly limit the amount of drug reaching the target tissue in an already difficult treatment site like the brain (Danhier et al., 2012). Nance et al. demonstrated that FUS-mediated BBB opening allowed delivery of PLGA-PEG particles to the brain within the focal region (Nance et al., 2014), indicating that FUS-mediated BBB opening may be leveraged to increase the probability that efficacious dosage levels of PLGA nanoparticles are met.

#### Poly(Aspartic Acid) (PAA)

**Figure 5:**
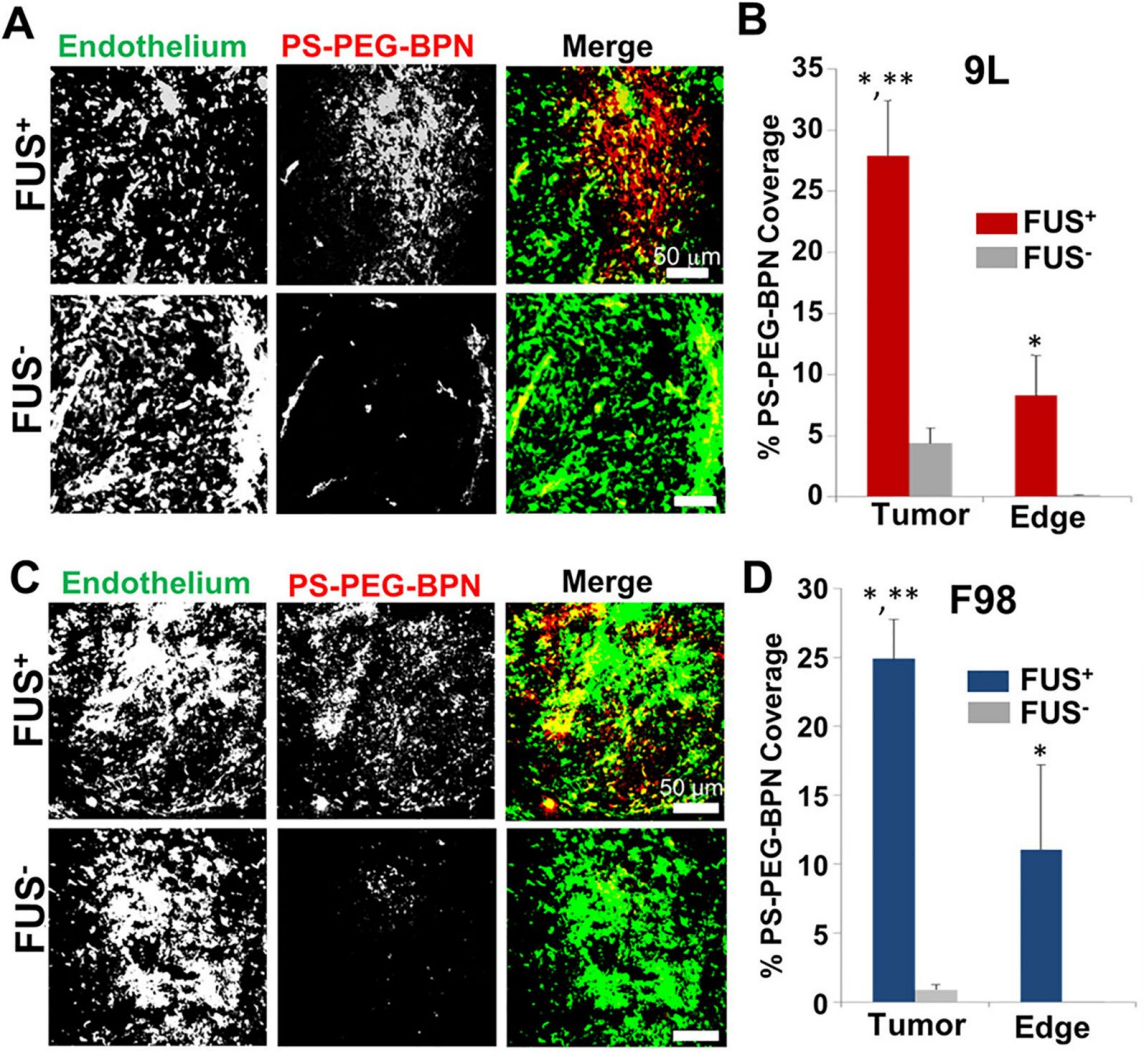
MR image-guided FUS markedly enhances the delivery of 60 nm fluorescent tracer nnaoparticles (PS-PEG-BPN) across the blood-tumor (BTB) and blood–brain barriers (BBB) in 9L and F98 tumors in rats. **(A)** Representative confocal microscopic images of 9L tumor cross-sections from FUS treated (FUS+) and untreated (FUS−) rats. PS-PEG-BPN (red) are shown in relation to tumor endothelium (green). **(B)** Bar graph of PS-PEG-BPN delivery to 9L tumors and tumor edge regions. N = 6 per group. *P < 0.05 vs. FUS− in same region. **P < 0.05 vs. FUS+ in Edge region. **(C)** Representative confocal microscopic images of F98 tumor cross-sections from FUS treated (FUS+) and untreated (FUS−) rats. PS-PEG-BPN (red) are shown in relation to tumor endothelium (green). **(D)** Bar graph of PS-PEG-BPN delivery to F98 tumors and tumor edge regions. N = 4 per group. *P < 0.05 vs. FUS− in same region. **P < 0.05 vs. FUS+ in Edge region. Adapted from Timbie et al. (2017). Reproduced with permission.

Though less commonly used, poly(aspartic acid) (PAA) nanoparticles are biodegradable nanocomplexes that offer higher drug-loading capacity. These nanoparticles have displayed the capacity to act as safe and efficient carriers for gene delivery (Nie et al., 2015; Song et al., 2015), as well as for drug delivery (Zhang et al., 2017). For example, using convection enhanced-delivery, an administration method which directly injects a substance into the brain, it has been demonstrated that PAA-PEG nanoparticles loaded with the chemotherapeutic cisplatin were able to diffuse through the brain parenchyma and increase survival time of rats with glioblastoma (Zhang et al., 2017). In another study, FUS administration drastically augmented delivery of polymer nanoparticles with a comparable PEG coating in two glioma models (Timbie et al., 2017) (**Figure 5**). After validating the ability of FUS to enhance nanoparticle delivery to brain tumors, the drug-loaded PAA-PEG nanoparticles were combined with FUS-mediated BBB opening to yield a markedly enhanced distribution of these PAA-PEG nanoparticles and a decrease in tumor growth (Timbie et al., 2017). Additionally, in this study Timbie et al. found that tumors treated with the cisplatin-loaded PAA-PEG nanoparticles and FUS displayed reduced invasiveness into surrounding tissue, suggesting this treatment may also inhibit glioma recurrence. These studies highlight the capability of PAA nanoparticles to be used in conjunction with FUS to deliver drugs and gene therapies to the brain, and this remains a rich avenue for therapeutic development in the future.

#### Polyethylenimine (PEI)

Developing nanoparticles for effective gene therapy as an alternative to viral vectors is an expanding area of research. Polyethylenimine (PEI) is a nanoparticle material commonly used for gene delivery (Lungwitz et al., 2005; Pandey and Sawant, 2016). Composed of many amine groups, PEI can bind compactly with DNA, and its free nitrogens are able to absorb protons within the acidic endosomal environment upon nanoparticle uptake (Behr, 1994; Kircheis et al., 2001). This proton absorption both mediates the disruption of the endosome and delays lysosomal fusion to the endosome (Godbey et al., 1999; Jere et al., 2009). Additionally, PEI facilitates transport into cell nuclei, mediating expression of the encapsulated gene (Godbey et al., 1999; Huang et al., 2010). Though often considered the gold standard of nanoparticle materials for gene transfection, PEI can demonstrate cytotoxicity (Godbey et al., 2001; Ira et al., 2003; Moghimi et al., 2005). However, when densely coated with PEG, PEI nanoparticles do not exhibit significant toxic effects to cells (Huang et al., 2010; Mastorakos et al., 2015; Mead et al., 2017). When administered *in vivo via* convection enhanced delivery, PEGylated PEI nanovectors carrying a reporter gene were able to transfect both healthy rat brain tissue and gliomas (Negron et al., 2019). Increased PEGylation resulted in a larger volume of transgene expression and greater percentage of the tumor volume. These PEGylated PEI nanoparticles also proved efficient for gene transfection when used with FUS to deliver a neurotrophic factor to the striatum of Parkinsonian rats (Mead et al., 2017). These studies bolster PEGylated PEI nanoparticles as a promising tool for FUS-mediated gene therapy in the CNS.

#### Poly(B-Amino Ester) (PBAE)

One biodegradable polymer that has received considerable recent interest as a nanoparticle component is poly(β-amino esters) (PBAE). Indeed, PBAE-based nanocomplexes have shown effective drug delivery in *in vitro* systems and are beginning to show promise *in vivo* (Green and Kim, 2019; Guerrero-Cázares et al., 2014; Mangraviti et al., 2015; Mastorakos et al., 2017). The potential for this nanoparticle was highlighted when its pH-sensitive solubility properties were utilized to release chemotherapeutics once in the decreased-pH endolysosomal environment (Shenoy et al., 2005). PBAE nanoparticles have now been used in the targeting of *in vivo* glioblastoma primary brain tumors. Using convection-enhanced delivery, it has been shown that DNA-loaded PBAE nanoparticles provide effective gene transfection of brain tumors in rats (Mastorakos et al., 2017). We affirm that the ability to combine PBAE nanoparticles with FUS delivery will greatly improve their ability to be used in more therapeutic applications and reduce the invasiveness of their administration. Moreover, PBAE particles have been shown to be robust. They have the capacity to be lyophilized and stored for up to two years and still display effective delivery to glioblastoma cells (Guerrero-Cázares et al., 2014). Going forward, one challenge in the design for FUS-compatible PBAE nanoparticles is making formulations that are stable in the bloodstream.

### Lipid-Based Nanoparticles for Delivery to the CNS With FUS

#### Liposomes

Liposomes have also been explored for brain-targeted drug delivery in conjunction with FUS-induced BBB opening (Treat et al., 2012; Yang et al., 2012). The main constituents of liposomes are amphiphilic phospholipids that form concentric bilayers (Puri et al., 2009). The aqueous core of the liposome can be loaded with hydrophilic or polar molecules, whereas the fatty acyl chains of the liposome bilayer can store hydrophobic molecules. The phospholipids that compose the liposome determines stability, loading efficiency, and physical phase. Commonly, chemically modified phosphatidylcholines are the primary phospholipid within a liposome. Frequently used modifications include dipalmitoylphosphatidylcholine (DPPC), hydrogenated soybean phosphatidylcholine (HSPC), dimyristoylglycerophosphatidylcholine (DGPC), distearoylglycerophosphatidylcholine (DSPC), and dioleoylglycerophosphatidylcholine (DOPC) (Chang and Yeh, 2012). Similar to nanoparticles, liposomes benefit from PEGylation to evade the reticuloendothelial system and absorption of blood proteins. PEG is typically introduced into liposomes *via* PEGylated lipopolymers like PEG-distearoylglycerophosphoethanolamine (DSPE) (Schroeder et al., 2009). Additionally, cholesterol is often utilized in the liposome composition to stimulate dense packing of the surrounding phospholipids, which enhances the liposome’s stability and decreases its permeability (Bozzuto and Molinari, 2015).

Liposome encapsulated drugs for brain delivery with FUS have been used with many drugs and for various applications (Treat et al., 2012; Yang et al., 2012; Lin et al., 2015; Lin et al., 2016; Zhao et al., 2018; Lin et al., 2019). In 2012, liposomal doxorubicin delivery to rat gliosarcoma following FUS-mediated BBB opening was investigated (Treat et al., 2012). Weekly examination of tumor growth *via* magnetic resonance imaging indicated that the combination therapy group of liposomal doxorubicin with FUS-mediated BBB opening slowed tumor growth compared to liposomal doxorubicin or FUS only groups. Further, the combination group also had a 24% increase in survival time over the nontreated group. More recently, liposomal glial-derived neurotrophic factor (GDNF) and FUS-induced BBB opening were used for treatment in a mouse model of Huntington’s disease (Lin et al., 2019). Mice were treated with either liposomal GDNF, BBB opening *via* FUS, or the combination of the two on a weekly basis for a total of 9 weeks and motor function was assessed during this time. Six weeks following the last treatment, mice were sacrificed, and brains were assessed for GDNF expression, protein aggregates, apoptosis, and downstream targets of GDNF. The combination of FUS-induced BBB opening and liposomal GDNF resulted in improved motor function compared to either treatment alone. Mice receiving the combination therapy had higher levels of GDNF expression, decreased protein aggregates and cell death, and increased neuron growth. These studies indicate the utility of FUS for greatly improving the therapeutic effects of drugs for brain-based delivery when encapsulated in liposomes.

#### Nanoemulsions/Nanodroplets

Phase-changing nanoemulsions or nanodroplets have recently gained interest for use with FUS to control spatial and temporal delivery of therapeutics within the brain (Chen et al., 2013; Wu et al., 2018; Yildirim et al., 2019). While these nanoparticles can be comprised of lipids or polymer shells, they have historically been encased *via* lipid-based bilayers (Yildirim et al., 2019). When administered, these nanoemulsions are composed of a lipid or polymer surface that encapsulates a liquid core and the therapeutic agent. Upon exposure to the FUS pressure waves, the liquid core transitions to gas. This expansion can eject and release the drug from the nanoemulsion at the focal site as well as be utilized to produce on-demand microbubbles. Nanoemulsions can be tricky to design, however, as instability for storage purposes and liquid-to-gas transition upon injection are common challenges for these particles (Rapoport et al., 2011). Careful considerations for boiling point of the nanoemulsion core, emulsifying agent, and needle gauge can help overcome some of these issues (Airan et al., 2017; Gorick et al., 2019).

One group indicated the feasibility of these particles for use with FUS to deliver molecules to the brain by targeting mouse hippocampus with dextran-loaded nanodroplets (Chen et al., 2013). Delivery with nanodroplets resulted in a more uniform delivery of dextran throughout the hippocampus when compared to microbubbles of the same lipid composition. This group later examined different liquid cores to optimize the delivery of molecules to the brain with FUS (Wu et al., 2018). Octafluoropropane and decafluorobutane-based nanodroplets were assessed for their vaporization efficiency and their ability to deliver dextran to mouse hippocampus. Octafluoropropane was found to have a greater vaporization efficiency, which lead to increased delivery of dextran to the brain under FUS application. Nanoemulsions offer an exciting new method to encapsulate molecules for brain-targeted delivery that can aid in temporal control in addition to the spatial control of FUS-mediated BBB opening.

#### Conjugated NP and Microbubbles

Up to this point, the nanoparticles discussed have been assumed to be injected separately from the microbubbles (i.e. nanoparticles unbound to microbubbles) upon application for drug delivery with FUS. Conjugating nanoparticles onto microbubbles has been explored as an option to increase delivery efficiency of the encapsulated agent. Nanoparticles can be bound to microbubbles *via* biotin/avidin interactions or—more commonly for *in vivo* studies—maleimide/thiol linkage (Mullin et al., 2013). Attachment of nanoparticles to microbubbles allows validation of nanoparticles’ presence at the site of BBB opening. Further, conjugation of nanoparticles to microbubbles enhances drug delivery by increasing cavitation near the nanoparticle (Schroeder et al., 2009). For the treatment of rat glioma, one group loaded liposomes with shRNA for targeting neovascular cells and conjugated these nanoparticles onto lipid-shelled microbubbles (Zhao et al., 2018). Rats receiving treatment of FUS and nanoparticle-microbubble complexes displayed decreased tumor growth and increased survival time than controls or individual (i.e. FUS or nanoparticle-microbubble complex only) treatment groups. Conjugated nanoparticle-microbubble complexes when combined with FUS-mediated BBB opening is a promising strategy for further increasing delivery efficiency in the CNS.

## Applications of Nanoparticles in Combination With Focused Ultrasound for the Treatment of CNS Pathologies

The use of nanoparticles for treating diseases has been investigated at the pre-clinical level for many years now. Recent developments in the use of FUS for safely opening the BBB have exposed new opportunities for nanoparticles in the treatment of neurological disorders. The following section discusses emerging developments of FUS-mediated nanoparticle delivery to the brain that have centered on designs allowing gene transfer, molecular targeting, and temporal control.

### Gene Therapy to the CNS With FUS

Gene therapy has gained considerable traction as a treatment option for many disorders, those of the CNS being no exception (Ojala et al., 2015; Keeler et al., 2017; Piguet et al., 2017; Price et al., 2019). Design for gene delivery vectors must balance between lowering cytotoxicity and increasing transfection efficiency. While viral vectors have often been the choice gene carrier, concerns remain about their loading capacity, safety, and production scalability (Thomas et al., 2003; Xu and Anchordoquy, 2011). Thus, non-viral vectors represent attractive alternatives for many applications, though they are not lacking in limitations of their own: decreased transfection efficiency, electrostatic interactions, and aggregation (Thomas et al., 2003; Mansouri et al., 2004; David and Doherty, 2017). Nanoparticles, many derived from formulations described above, have been coupled with FUS to deliver genes as a therapeutic treatment for neurological disorders (Suzuki et al., 2011; Timbie et al., 2015).

In 2014, Nance et al. were amongst the first to characterize the ability of FUS to deliver systemically-administered polymer nanoparticles to magnetic resonance image-targeted brain regions (Nance et al., 2014). Using simple fluorescent polystyrene (PS)-PEG tracer nanoparticles, the authors characterized the effect of nanoparticle size on diffusion within *ex vivo* and *in vivo* rat brains. Compared to 110 nm and 240 nm particles sizes, 60 nm particles had the least hindered transport rate. When systemically injected and delivered across the BBB with FUS, these 60 nm PS-PEG nanoparticles penetrated the brain parenchyma, and their coverage increased with increasing FUS pressures. Additionally, they validated that this FUS-mediated delivery to targeted brain regions could be extended to a 75 nm, biodegradable PLGA-PEG nanoparticle.

**Figure 6:**
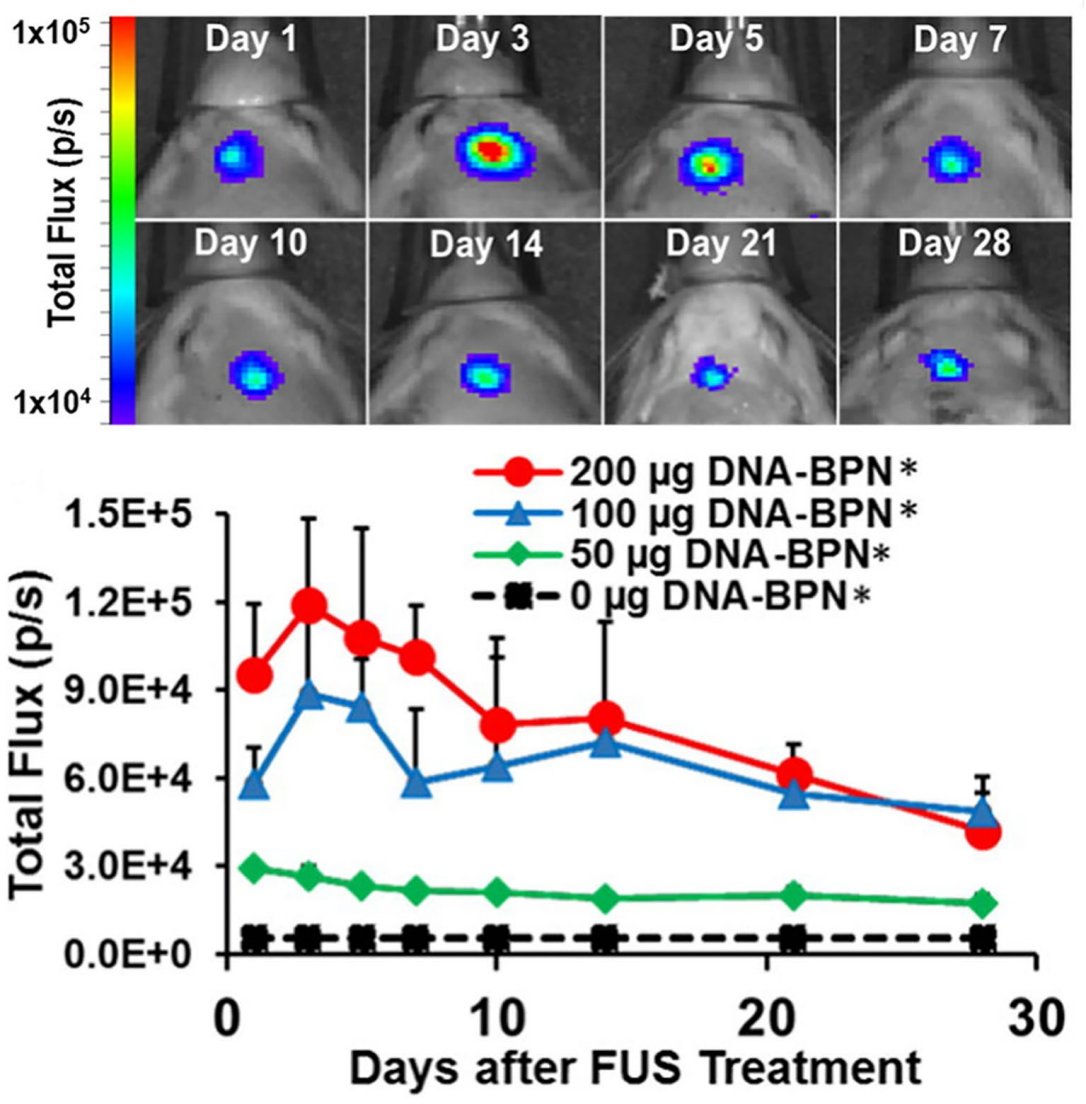
FUS-mediated delivery of reporter gene-bearing non-vial nanoparticles (DNA-BPN) across the BBB leads to robust and localized transgene expression in the rat brain. Top: Representative IVIS bioluminescence images after DNA-BPN delivery to rat brain using FUS and MBs. Bottom: Line graph of bioluminescence total flux over the 28-day test period. n = 5 at each dose. *Significantly different than all other doses tested (p < 0.05). Adapted from Mead et al. (2016). Reproduced with permission.

Moving this work into gene therapy, Mead et al. used highly compacted (56 nm) PEI-PEG nanoparticles to transport a luciferase reporter gene under a β-actin promoter to rat striatum (Mead et al., 2016). After FUS-mediated BBB disruption and systemic injection of the gene-loaded nanoparticles, bioluminescence—indicating transfection of the luciferase reporter gene—was visible only within the focal region targets and persisted for at least 28 days as detected by *ex vivo* bioluminescence (**Figure 6**). Of note, bioluminescent signal could be detected within a day with these non-viral vectors in contrast to longer delays that may be seen with some viral vectors (Miao et al., 1998).

**Figure 7:**
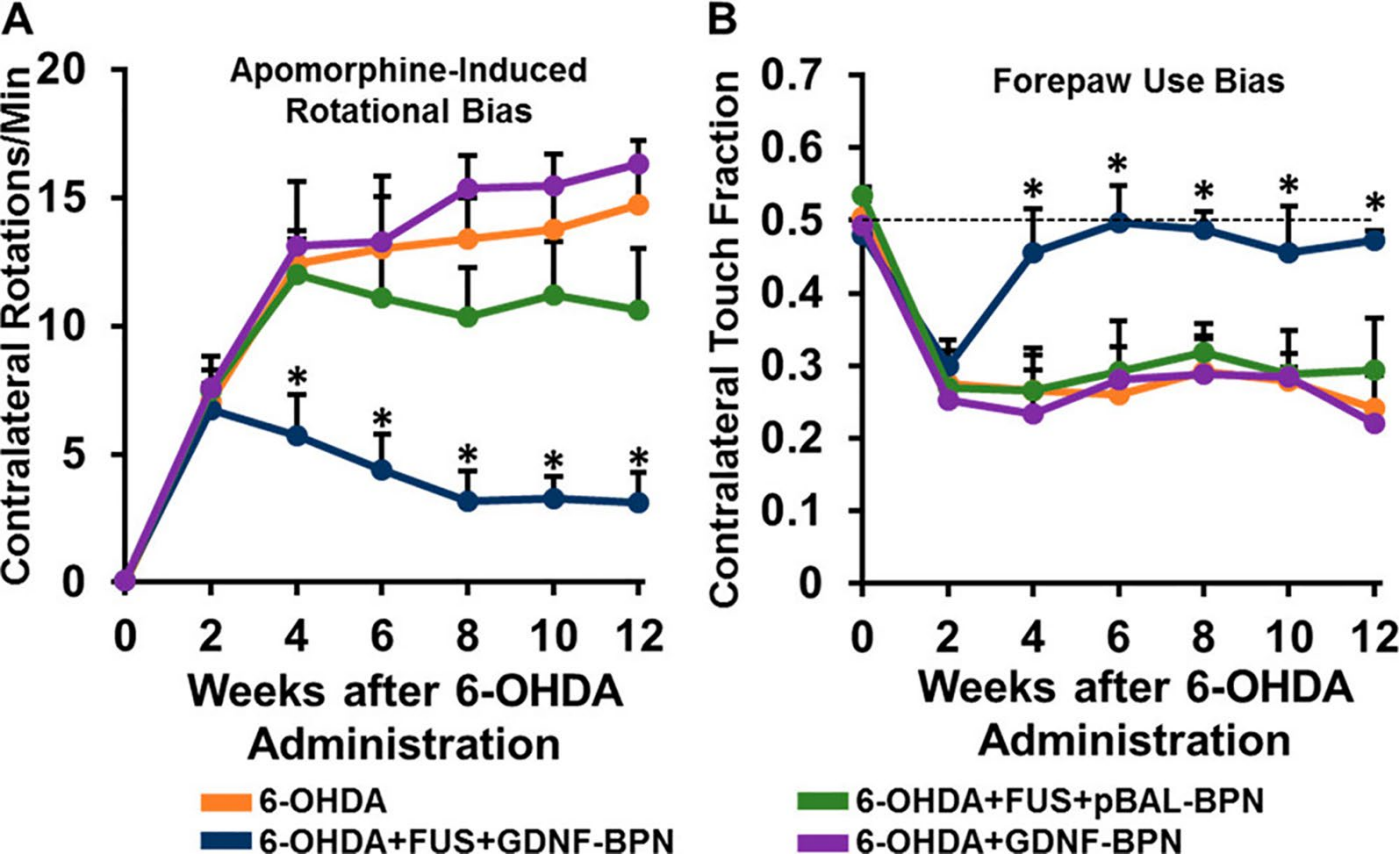
Delivery of glial cell-derived neurotrophic factor gene-bearing nanoparticles (GDNF-BPN) with FUS restores locomotor function in PD rats. **(A)** Line graph of average contralateral rotations per minute after apomorphine administration. **(B)** Line graph of contralateral touch fraction in the forepaw use bias test. n > 14 in each group at weeks 0 through 6; n > 7 in each group at weeks 8 through 12. *Significantly different than all other groups at the same time point (p < 0.01).Adapted from Mead et al. (2017). Reproduced with permission.

Implementing these findings to CNS disorders, the same group successfully used this FUS-mediated gene therapy delivery system to reverse Parkinsonian behavioral and molecular deficits in a neurotoxin-induced, rat model of Parkinson’s disease (Mead et al., 2017). PEI-PEG nanoparticles carrying a plasmid for GDNF were intravenously injected, and FUS was applied to the neurotoxin-induced lesion of the left striatum. Twelve weeks following GDNF delivery, both striatal GDNF and dopamine levels were significantly elevated in the treated striatum compared to those of the neurotoxin-only control rats. Beginning at week 4 and extending until at least week 12, behavioral deficits were also rescued in the FUS and GDNF-treated rats as measured by the apomorphine-induced rotational bias and forepaw use bias behavioral tests (**Figure 7**). These results indicate that FUS-mediated BBB opening is an auspicious method in the application of gene therapy for the treatment of neurological disorders.

Similarly, another group demonstrated the ability to use a PEGylated liposome of DPPC/cholesterol composition to encapsulate either luciferase reporter gene or GDNF. They then used FUS to deliver this therapeutic liposome into the right hemispheres of healthy mice (Lin et al., 2015). Luciferase expression was maintained for at least 4 days following FUS treatment and was significantly higher than non-encapsulated luciferase. Additionally, the authors found that gene expression was dose dependent; however, the day of peak expression differed for the different doses. FUS-induced BBB opening and delivery of liposomal GDNF plasmid was able to increase GDNF expression over control group, whereas liposomal GDNF plasmid administration did not. These studies indicate the promise of gene delivery through a variety of nanoparticle formulations in CNS disorders, further potentiated by the ability to overcome the BBB noninvasively with FUS-mediated delivery (Price et al., 2019).

### Molecular Control of Drug Delivery to the CNS With FUS

The capacity to use FUS to selectively administer a therapeutic agent to a specified brain region in itself creates promising therapeutic opportunities never before available. To go beyond the spatial targeting that FUS allows, applying this system with nanoparticles that have receptor-specific ligands conjugated to their surface can allow molecular targeting within a defined region. This combination opens the door for treatments that require targeting of particular receptors but only in a specific brain region.

Luo et al. took advantage of the fusion of FUS-mediated BBB opening and molecular-targeting nanoparticles to treat glioblastoma. Both the endothelial cells lining the BBB and glioblastoma cells are abundant in lipoprotein receptor-related protein, which binds the ligand angiopep-2 (Luo et al., 2017). Angiopep-2-conjugated PLGA nanoparticles were designed to release encapsulated doxorubicin and perfluorooctyl bromide upon FUS exposure. PLGA nanoparticles with angiopep-2 surface modifications specifically accumulated in glioblastoma cells at more than 13-fold higher than the same nanoparticle without angiopep-2 conjugation. Another group compared the ability of liposomal doxorubicin and angiopep-1-conjugated liposomal doxorubicin to target and accumulate in murine glioblastoma when combined with FUS-mediated BBB opening (Yang et al., 2012). They found that FUS+peptide-conjugated liposomes had a significantly higher uptake in the brain tumors than FUS+non-targeted liposomes, and both groups had a higher uptake than either liposome without FUS-induced BBB disruption. These results of increased targeting of glioblastoma cells highlights the utility of combining FUS with molecular-targeting nanoparticles in the CNS. Moreover, in many diseases of the brain there are molecular targets unique to the disease that could be utilized in nanoparticle fabrication. For Alzheimer’s disease, nanoparticles have already been designed to target amyloid beta aggregates (Song et al., 2016). Combining molecular delivery of molecular-targeting nanoparticles following FUS-mediated BBB opening could hold potential for greatly improving drug delivery to CNS disease targets.

### Temporal Control of Drug Delivery to the CNS With FUS

**Figure 8:**
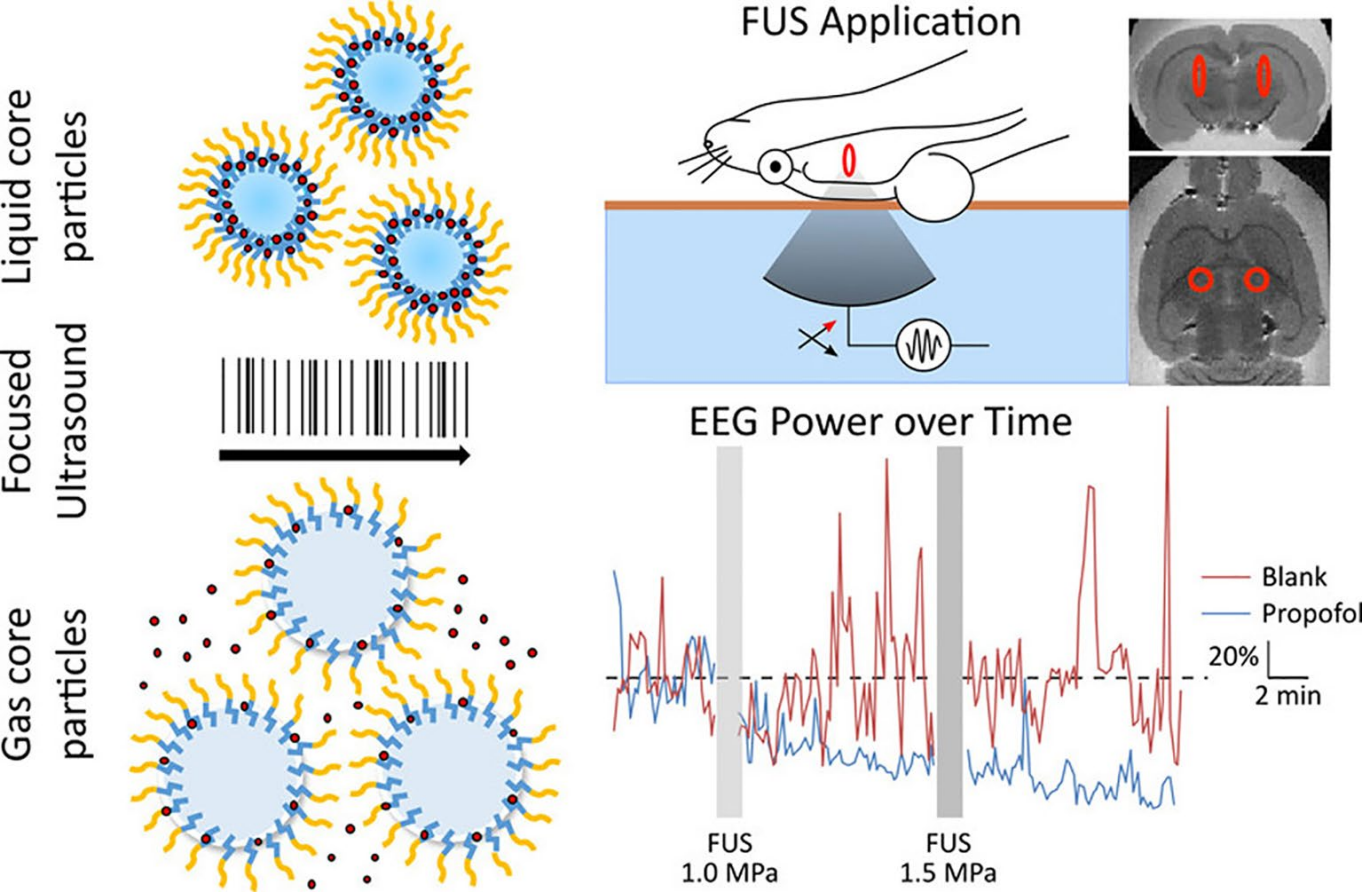
Liquid nanoparticles composed of biodegradable and biocompatible components and containing propofol release the drug upon exposure to focused ultrasound. The uncaged propofol was shown to silence seizures in an acute rat seizure model. Adapted from Airan et al. (2017). https://pubs.acs.org/doi/abs/10.1021/acs.nanolett.6b03517. Reproduced with permission. Further permissions related to this figure should be directed to the American Chemical Society (ACS).

In addition to molecular targeting, nanoparticles can also be designed to be responsive to pressure or temperature changes. Using FUS to “trigger” the release of drugs from these nanoparticles adds a new mechanism of temporal control to this drug delivery system. As discussed previously, one unique category of pressure-sensitive nanoparticles that have been used with FUS for brain-targeted delivery are nanoemulsions. In application, these nanoemulsions provide a temporal mechanism for drug delivery with release only occurring throughout the duration of FUS. FUS-activated nanoemulsions loaded with Propofol are a noteworthy example (Airan et al., 2017) (**Figure 8**). While the drug Propofol is already capable of bypassing the BBB, normal systemic administration of the anesthetic does not allow for spatial targeting within a certain region of the brain, leading to a dysregulated, anesthetic effect. FUS in this application was not used to open the BBB but rather to trigger drug release at the desired brain region. After inducing convulsions in a rat seizure model, FUS was applied to release Propofol to a select brain region in both hemispheres. As validated *via* electroencephalogram, FUS-mediated, targeted Propofol release suppressed seizures in these rats. This application is particularly promising in the case of neuromodulation. While repeated BBB opening with FUS will be clinically acceptable in the setting of many debilitating CNS diseases, it will likely be contraindicated for others. These low-intensity, nanoemulsion activation approaches may be especially useful in such applications. Further, in the case of psychiatric disorders, many drugs have already been developed that are capable of bypassing the BBB but require localized administration to increase their efficacy and reduce side effects. These FUS-activated nanoemulsions have demonstrated the capability of encapsulating other hydrophobic drugs which gives promise to the extension of this application with other psychiatric medications (Zhong et al., 2019).

Another emerging, pressure-sensitive nanoparticle is gas vesicles. Gas vesicles are derived from photosynthetic microbes that produce these gas-filled nanostructures to change their buoyancy for optimal light exposure (Lu et al., 2018). By responding to magnetic fields dissimilarly than water, these gas vesicles can act as magnetic resonance image contrast agents. Using FUS, gas vesicles can then be collapsed to produce background images that can be subtracted from the intact gas vesicle images. This background image subtraction enhances the signal-to-noise ratio, which is especially helpful in scenarios with confounding background signals, such as is the case with some tumors. Because these are biologically produced nanostructures, gas vesicles can be genetically modified to respond to different pressures. This feature allows for the construction of multiplexed magnetic resonance images. In bacterial hosts, gas vesicles can be expressed under inducible promoters. Extension of this inducible expression into mammalian hosts holds exciting promise for capturing changes in molecular states or cellular responses *via* non-invasive magnetic resonance imaging.

The ability of FUS to be applied and specifically heat tissue allows another application of nanoparticle design: thermosensitive nanocomplexes. Magnetic resonance image-guidance, in addition to providing precise targeting, lends access to monitoring changes in temperature to the tissue—a technique known as magnetic resonance-thermometry. Nanoparticles that are sensitive to these heat changes allow drug release to occur once the target temperature is reached. In the case of tumor treatments, thermosensitive nanoparticles are particularly desirable as tumor ablation can be paired with drug release. In one study, treatment with thermosensitive liposomes that encapsulated the chemotherapeutic doxorubicin prior to tumor ablation delayed tumor growth to a greater extent than either treatment alone (Hijnen et al., 2017). Another study indicated that PEG-coated liposomal encapsulation of doxorubicin combined with FUS-induced hyperthermia successfully constrained tumor growth in a murine brain metastasis model following just a single treatment (Wu et al., 2014).

## Diagnostic and Functional Testing Applications of Nanoparticles and FUS

The use of FUS with nanoparticles for the treatment of CNS disorders is exciting and propitious for the development of new therapeutic options in this challenging field. Nevertheless, the noninvasive nature of this technology also holds great potential for its applications where only minimal perturbation of a subject is warranted. Aside from therapeutic applications of FUS and nanoparticles, this combination can also be implemented for disease screenings and functional studies of the brain.

### Detection and Diagnosis

With few standard screening processes in place currently, detection of neurodegenerative diseases is sorely needed (Visser et al., 2008; Panegyres et al., 2016). Reliant on the onset of their symptoms for diagnoses, these CNS diseases are often too far progressed to have effective treatment options. Similar to imposing surface modifications for specific drug release, molecular-targeting nanoparticles can also be leveraged to detect or enhance imaging of disease markers. The capability to fabricate nanoparticles that target disease markers and to utilize FUS to deliver them to the CNS is emergent. Using FUS to deliver antibodies across the BBB has been demonstrated several times, indicating the possibility to allow antibody-conjugated nanoparticles to bypass the BBB for detection of CNS disease markers (Sheikov et al., 2004; Kinoshita et al., 2006; Aryal et al., 2014). In the case of Parkinson’s disease, Alzheimer’s disease, and Huntington’s disease, the ability to detect early aggregation of their molecular signatures (alpha-synuclein, amyloid beta, and mutant huntingtin, respectively) could be a promising detection method (Manoutcharian et al., 2016).

### Functional Studies

Our ability to develop novel and more effective treatments for CNS disorders relies upon a continued commitment of understanding normal brain function. To this end, the ability to use FUS and nanoparticles to noninvasively perturb brain activity is a promising, unmatched method to investigate the functional connectivity of the brain. Using FUS-activated nanoemulsions carrying Propofol, select rat brain regions were targeted for local Propofol release, and then positron emission tomography (PET) imaging was used to assess global changes in brain activity (Wang et al., 2018). As optogenetic modulation of the frontal cortex has been shown to modulate activity in the thalamus, researchers investigated if targeted release of Propofol to the thalamus could thus elicit functional changes to the frontal cortex. Indeed, frontal cortical activity changes were seen upon uncaging of Propofol in the thalamus. As the thalamus regulates many brain regions, unsurprisingly, many other brain regions also showed altered function after thalamus targeting. This study is particularly exciting for functional studies of the brain in alive, minimally-perturbed animal models. Additionally, the ability to use FUS for neuromodulation comes with the advantage of being able to target deep brain regions which is not possible with optogenetic techniques that require transmission of light for activation.

## Conclusion

Despite many positive developments and significant pre-clinical progress, many debilitating CNS disorders still do not have cures. Further, in many cases, even symptomatic treatment options are limited. Combining non-viral nanoparticles that are capable of controlling pharmacokinetic mechanisms with FUS to spatially and noninvasively deliver therapeutics to the brain creates potentially powerful drug and gene delivery systems. Such combinations offer therapeutic options that are not possible with other technologies used for bypassing the BBB. Additionally, FDA-approved FUS systems for targeting of the brain already exist and are showing promising initial results for inducing BBB opening in clinical trials (Carpentier et al., 2016; Lipsman et al., 2018; Idbaih et al., 2019; Mainprize et al., 2019). Looking ahead, the capacity to administer biocompatible nanoparticles to mitigate otherwise toxic or adverse delivery of therapeutic agents with the additional advantage of controlling spatial, molecular, and temporal delivery of therapeutics to the brain noninvasively with FUS affirms the advantage of this combined approach for neurological treatments, detection, and functional studies.

## Author Contributions

Writing—Original Draft Preparation: DF. Writing—Review and Editing: DF and RP. Supervision: RP. Funding Acquisition: RP.

## Funding

This work was supported by NIH R01NS111102, R01CA197111, and R01EB020147.

## Conflict of Interest

The authors declare that the research was conducted in the absence of any commercial or financial relationships that could be construed as a potential conflict of interest.
